# Supplementary material for: Northern expansion is not compensating for southern declines in North American boreal forests
Source: Nat Commun. 2023 Jun 8;14:3373. doi: 10.1038/s41467-023-39092-2 (PMC10250320; doi:10.1038/s41467-023-39092-2)
Supplement: Supplementary file 1 — Supplementary information [file 41467_2023_39092_MOESM1_ESM.pdf]

Northern expansion is not compensating for southern declines in North American boreal forests

Supplementary information

First and corresponding author: Ronny Rotbarth

Co-authors: Egbert H van Nes, Marten Scheffer, Jane Uhd Jepsen, Ole Petter Laksforsmo Vindstad, Chi Xu, Milena Holmgren

Supplementary Discussion

We found that tree cover across the boreal biome increased in all sample plots (0.12% per year) as well as across the entire biome. This increase in tree cover constitutes an increase of the surface area covered by trees. This increase was mainly driven by an infilling of available space in the northern interior. Here, we demonstrate that such an area increase is consistent with a range shrinkage of geographical tree cover distribution. Such shrinkage is caused by the lack in tree cover expansion at the northern boreal boundary and a reduction in tree cover at the southern boundary. To that avail, we have used tree cover distributions along standardised boundary distances for the year 2000 from our sample plots to simulate tree presence. We created a raster of 500x500 pixels, whereby columns represent a generic width of forest and rows represent standardised boundary distances from -1.5 to 1.5. In each raster row (i.e. each standardised boundary distance), we filled the values with either '0' for tree absence or '1' for tree presence by drawing from a binomial distribution. The probability of this distribution was determined by the mean tree cover of each standardised boundary distance (e.g. tree cover at -1, the southern boreal biome boundary, averaged 42.7%, so we used a probability of 0.427 in a binomial distribution). This way, we created a raster in which the proportion of trees for each standardised boundary distance approximates the observed tree cover (Figure 1a). We then calculated tree cover for the year 2019 in the following way:

$$\text{Tree cover}_{2019} = \text{Tree cover}_{2000} + 20\ \text{years} * \Delta\ \text{Tree cover}_{2000-2019},$$

where $\Delta\ \text{Tree cover}_{2000-2019}$ is the annual tree cover trend from 2000-2019. We did not use the remotely-sensed tree cover values for 2019, as the way we quantified tree cover trend accounts much better for the change across the entire 20-year study period than the single year 2019 and also accounts for the considerable variability in the data set. We then used the resulting tree cover in the same way as for the year 2000 and created a second raster representing the condition of tree presence in 2019 (Figure 1a).

We aimed at identifying a potential change in the distribution of tree cover. Therefore, we used the biome boundary distances (-1 for the south and 1 for the north) and calculated the quantiles of tree distributions at both locations using an empirical cumulative distribution function. The southern boundary is associated with the 26.5% and the northern boundary with the 97.6% quantile. The southern boundary is not closer to the lower quantiles of tree distribution because boreal forests transition to temperate forests across large parts of the southern biome. Hence, tree cover remains at relatively high levels. We used the quantiles from 2000 and identified the positions where the boundaries should be based on the same quantiles of tree distribution in 2019 (purple lines in Figure 1a). We observed a noticeable northward shift of the southern boundary and a slight southward shift of the northern boundary over the past 20 years. We interpret these shifts as a shrinkage of the distribution range of tree cover.

We finally compared the distribution of simulated tree cover between years (Figure 1b). For large parts of the boreal biome, especially the northern interior, tree cover has increased. We can convert tree cover into a unitless measure of area. The comparison of the total area sum of 2000 and 2019 confirms the

overall increase in tree cover we observed. Thus, we demonstrated the simultaneous occurrence of range contraction and area increase across North American boreal forests.

To provide further evidence for the possible onset of a biome contraction, we explored changes in tree cover distributions along the northern and southern boreal boundaries. A latitudinal shift and range shrinkage of tree cover distributions over time could be a strong indicator for an impending biome contraction. As shown above, trends in tree cover would lead to a northward shift of the southern distribution boundary and a stationary northern boundary. A resulting shrinkage would be expected to show the following additional signs:

(1) A shift in tree cover distributions from dense forests to open forests of lower tree cover along the southern boundary and a near stable tree cover distribution along the northern boundary.
(2) A mismatch between tree cover distribution changes along boundaries, characterised by considerable reductions in the proportions of high-density forests in the south and smaller reductions in the proportions of low-density forests in the north.
(3) A northward latitudinal displacement of high-density forests at the southern boundary which outpaces any equivalent displacement of low-density forests at the northern boundary.

In an attempt to identify above processes for the entirety of the North American boreal biome, we have extracted all MODIS tree cover data within a band of 120km on each side of the northern and southern boundary (Figure 2). As the year-to-year variation in the dataset is high, we have averaged tree cover for each data point for the first three years of the study period (2000-2002) and final three years (2017-2019). The total number of data points for each time period was 52,761,493 and 50,222,296 for the north and south, respectively.

We found evidence for all three processes above:

Shifts in tree cover distributions

We observed shifts along both boundaries consistent with a biome contraction. Along the northern boundary, the most notable shift occurred from low-density forests to near-zero tree cover (Figure 3a). While the distribution of tree cover did not remain stable, as expected, there was also no evidence of increases in tree cover at low densities which would indicate an expansion of forests. Along the southern boundary, tree cover distributions shifted as expected. Tree cover clearly decreased from a peak around 65% to around 55% (Figure 3b). Such a shift is expected based on the clear negative tree cover trends we observed in our sample plots. Here, we showed that this reduction in tree cover in the south is unmatched by any increases in tree cover at the northern boundary. As we used all data points around boundaries, this mismatch in distribution shifts also indicates a shrinkage of the tree-covered area of the boreal biome around the boundaries. The northern boreal lost around 450,000ha and the southern boreal around 2,600,000ha, i.e. a total loss of around 3,000,000ha. As such, we found evidence for a contraction in both tree cover distributions and area.

Mismatch between proportional changes in tree cover

Coupled with the observed shift in tree cover distributions around the boundaries, we found a clear mismatch between proportional changes in tree cover classes between the north and the south. Near-zero tree cover areas (0-5%) increased by around 50% in the north (Figure 4). Low tree cover areas declined by 50%. While we found a doubling of moderate tree cover between 20% and 40% in the north, these areas were far less frequent than low tree cover areas (compare with Figure 3a). In the south, we observed an almost 100% loss in areas of tree cover higher than 65% and a corresponding increase in the proportion of areas with tree cover between 30% and 60% (Figure 4). The loss in proportions of dense forests in the south outweighs the increases in proportions of low-density forests in the north.

Latitudinal displacement of tree cover

Besides a change in tree cover distributions indicative of a biome contraction, we also found a clear mismatch in latitudinal displacement of tree cover between boundaries. At the northern boundary, low tree cover densities have shifted northward by 1°-1.5° (Figure 5). This shift is outpaced by a northward displacement of high tree cover densities in the south of up to 4° (Figure 5). Consequently, the mismatch in latitudes in the north and south indicates a northward shift of the boreal biome based on tree cover. This shift is asymmetrical with a four times faster pace of the southern shift than of the northern shift.

In summary, we found multiple lines of evidence pointing towards the possible onset of a boreal biome contraction based on tree cover trends, shifts in tree cover distributions at the biome boundaries and latitudinal displacement of forests of different densities. Even though our study period is too short to show a complete loss of tree cover in the southern boreal biome, the clear forest thinning and contraction of tree cover distributions of the North American boreal biome points towards the long-term replacement of boreal forests, e.g. by temperate forests, shrublands or grasslands. We believe that the observed tree cover dynamics will continue into the future due to the connection of tree cover trends with disturbances and temperatures we observed. We see no reason for a reduction in magnitude of these drivers. In fact, a biome contraction may even accelerate with further climate change and increased disturbance pressure.

Supplementary figures

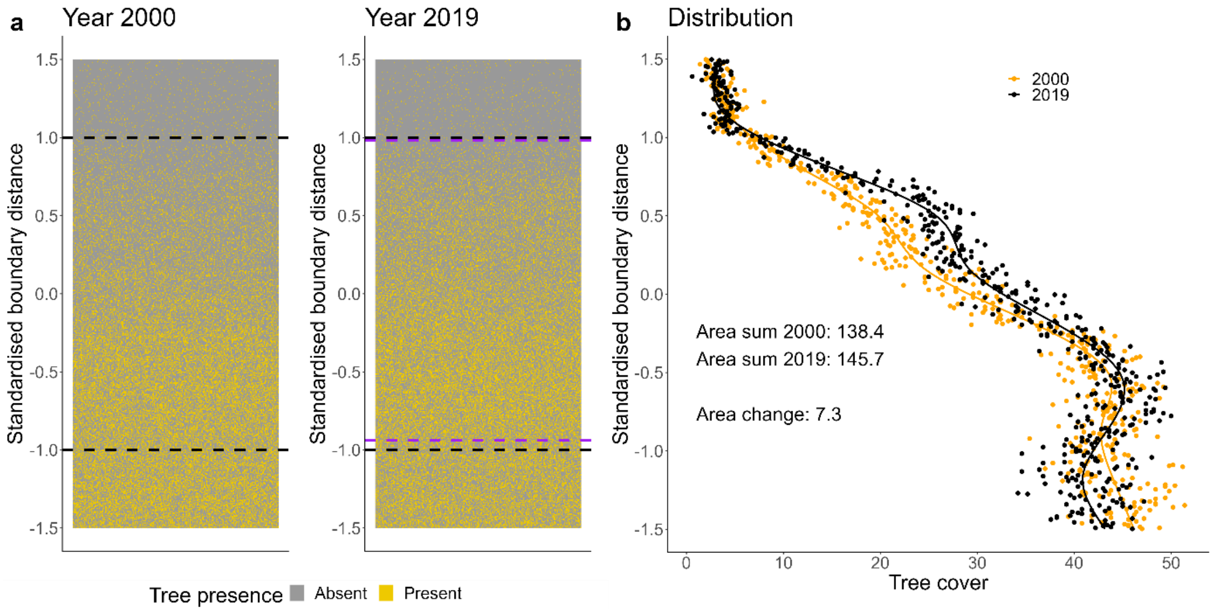

Figure 1 Simulated tree cover distribution and area change of North American boreal forests based on remote sensing time series. a Simulated geographical distribution range of tree presence for the years 2000 and 2019. Tree presence for the year 2000 was simulated through binomial distributions using actual mean tree cover observations along latitudinal gradients (i.e. standardised boundary distances). Tree presence for the year 2019 was simulated using observed tree cover trends over the entire 20-year period. Black horizontal lines represent the southern and northern boreal biome boundaries based on Gauthier *et al.* (2015)[1]. Purple lines show the position where these boundaries would be in 2019 based on the same tree distribution quantiles (see Supplementary discussion). **b** Tree cover for the years 2000 and 2019 and associated area change based on simulated data in (A).

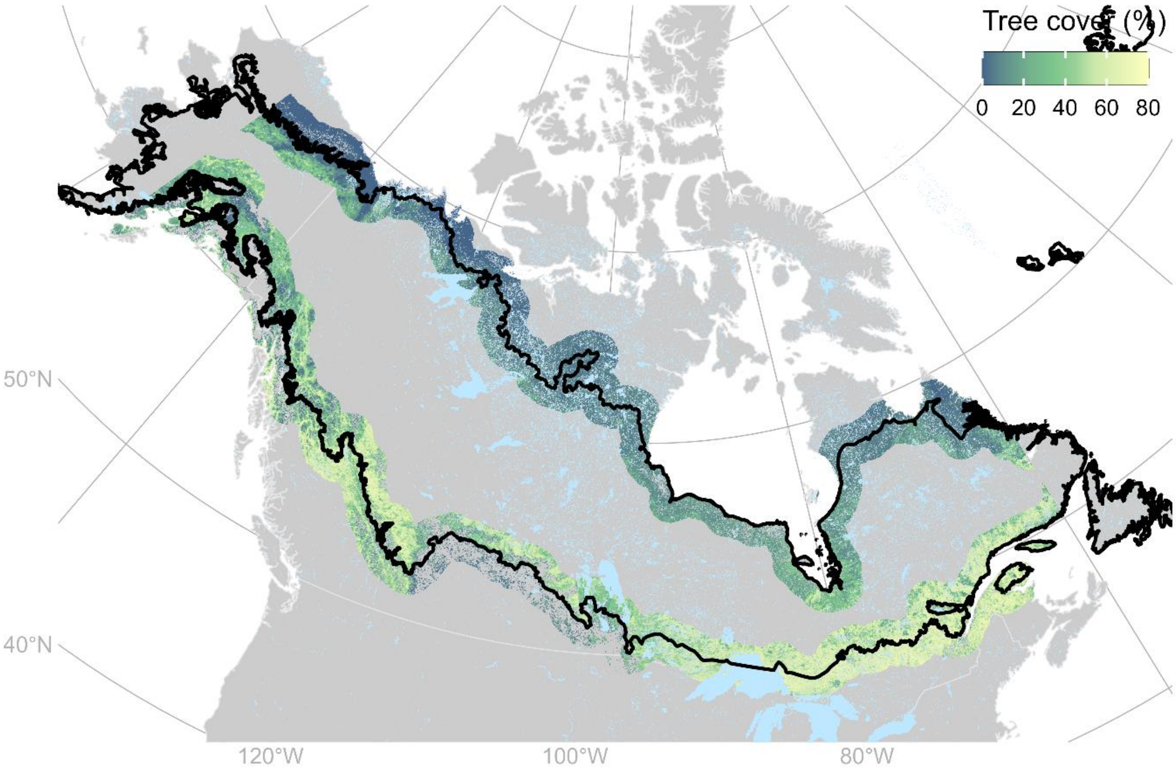

Figure 2 Tree cover around the southern and northern boreal biome boundaries. Tree cover is expressed as means for each data point for the years 2000-2002. The band around each boundary extends to both sides by 120km. Tree cover at the easternmost and westernmost boreal biome were excluded, as the biome boundaries curve towards each other which makes a distinction between boundaries impossible.

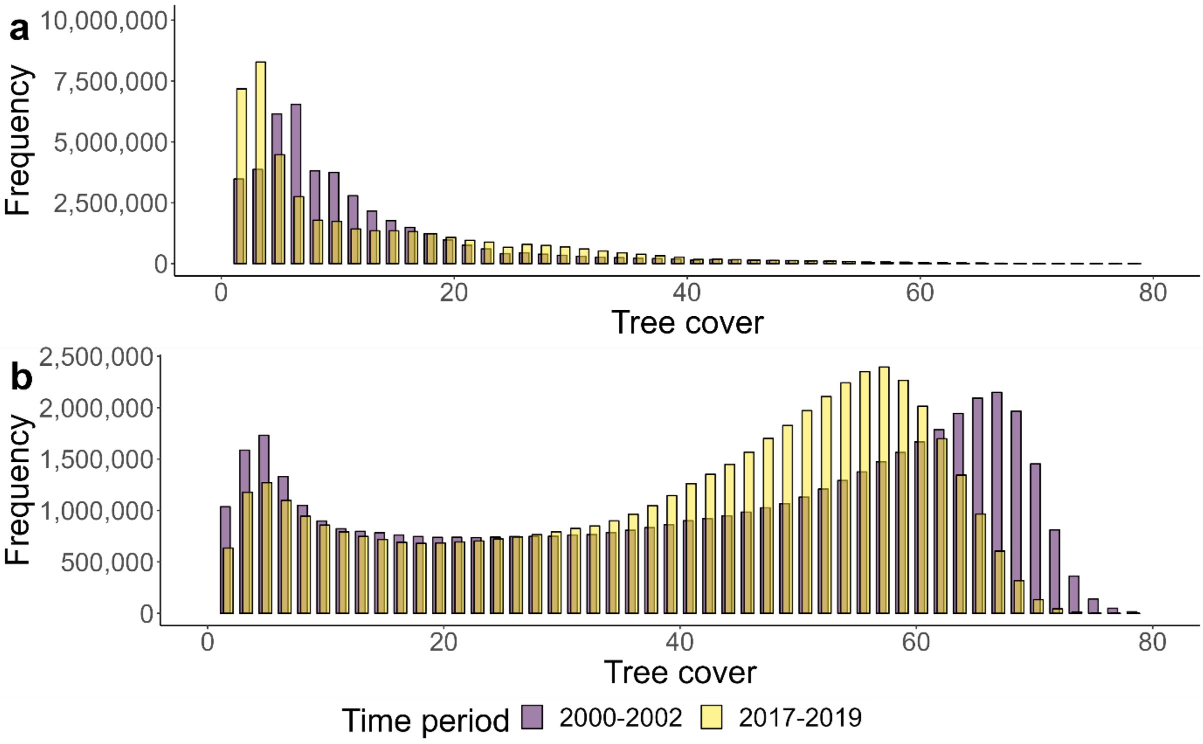

Figure 3 Changes in tree cover distributions of the northern and southern boreal biome. Distributions are shown for the northern (**a**) and southern (**b**) boundary between the periods 2000-2002 and 2017-2019.

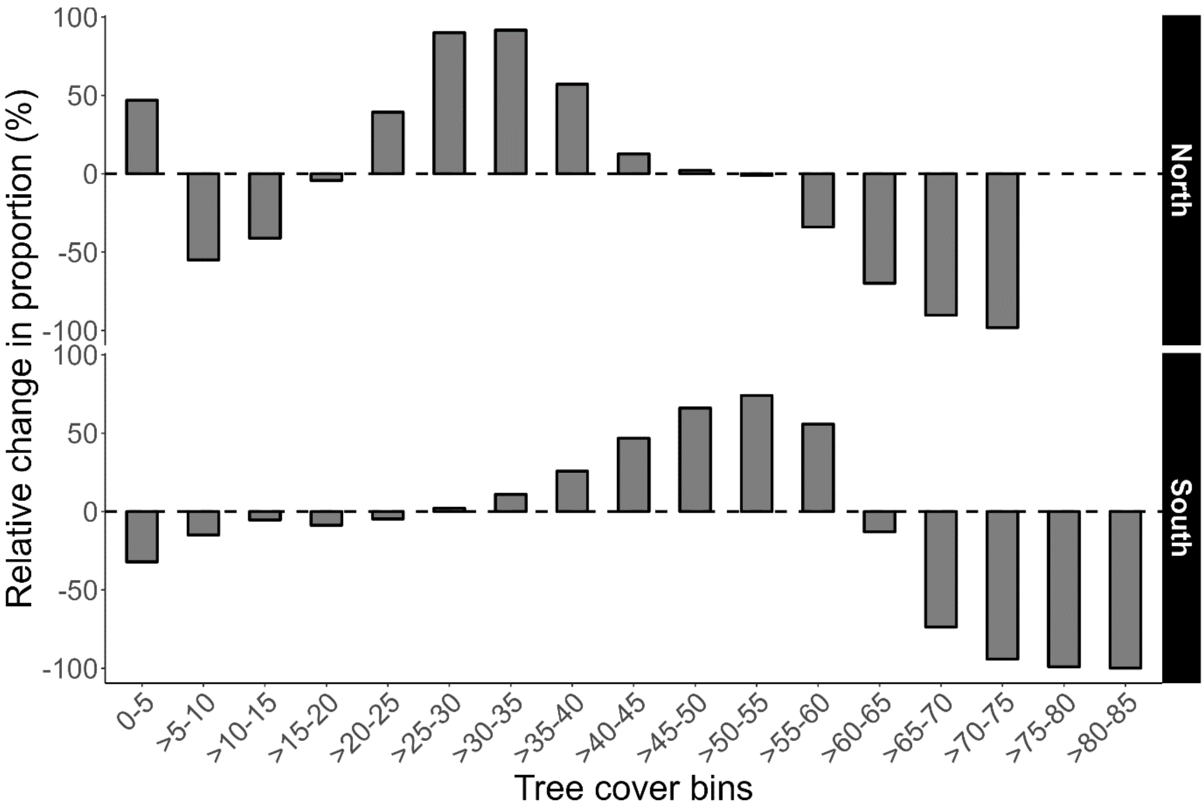

Figure 4 Changes in the relative proportion of tree cover classes along boreal biome boundaries. Changes are shown for the periods 2000-2002 and 2017-2019 and for the northern and southern boundary. Tree cover is expressed in bins of 5%. Change in proportions is expressed as relative change based on the proportions for the period 2000-2002.

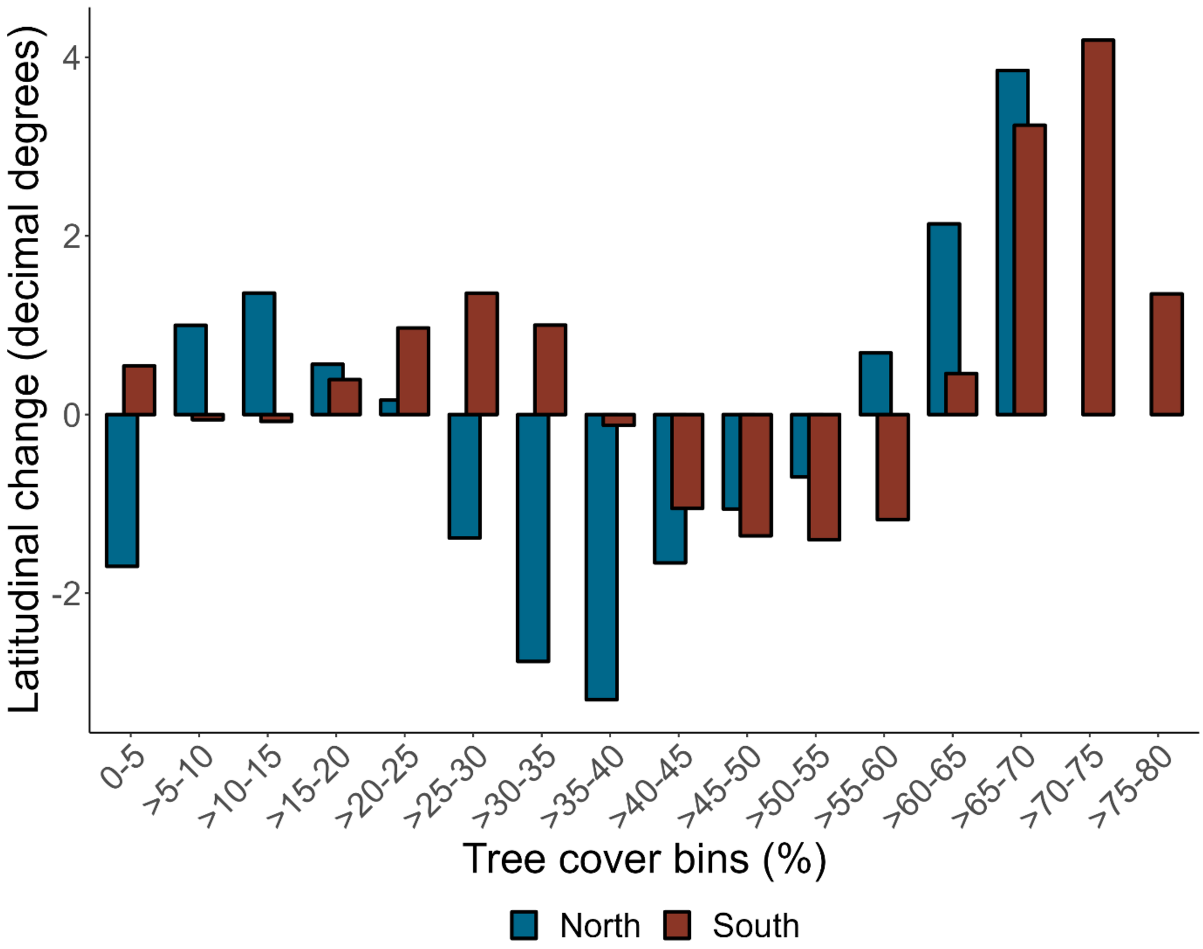

Figure 5 Latitudinal displacement of tree cover classes. Shifts are shown for the periods 2000-2002 and 2017-2019 along the northern and southern boreal biome boundary. Displacement is based on the mean change in latitudes of each tree cover class between periods. Tree cover classes are expressed in 5% bins.

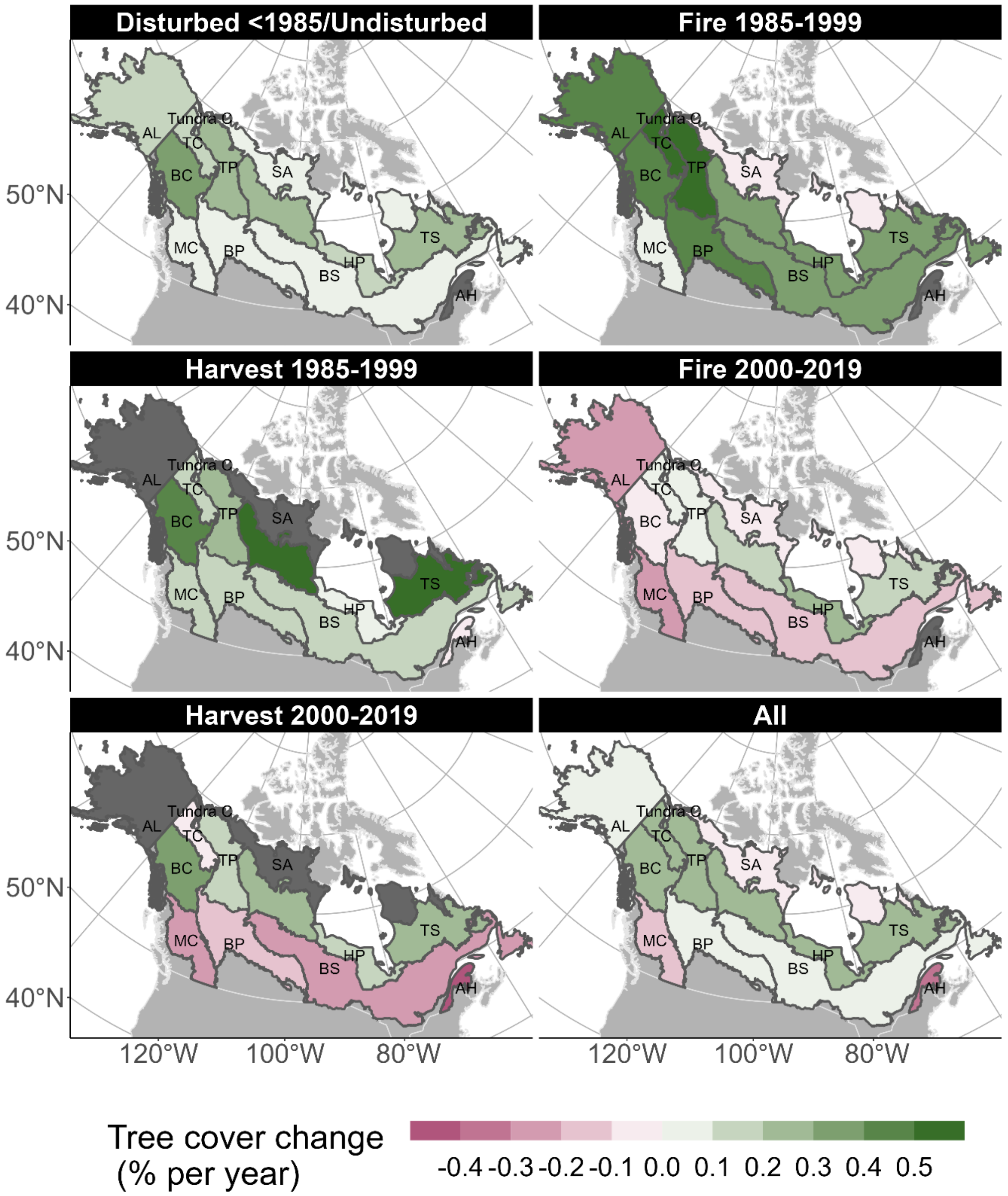

Figure 6 Mean tree cover changes 2000-2019 across Canadian ecozones and Alaska. Tree cover changes are shown for different disturbance types and timing. 'All' shows tree cover changes irrespective of disturbance. Tree cover changes were averaged based on sample plots within each ecozone. Zone labels: AL = Alaska, AH = Atlantic Highlands, BC = Boreal Cordillera, BP = Boreal Plain, BS = Boreal Shield, HP = Hudson Plains, MC = Montane Cordillera, SA = Southern Arctic, TC = Taiga Cordillera, TP = Taiga Plain, TS = Taiga Sield, Tundra C = Tundra Cordillera.

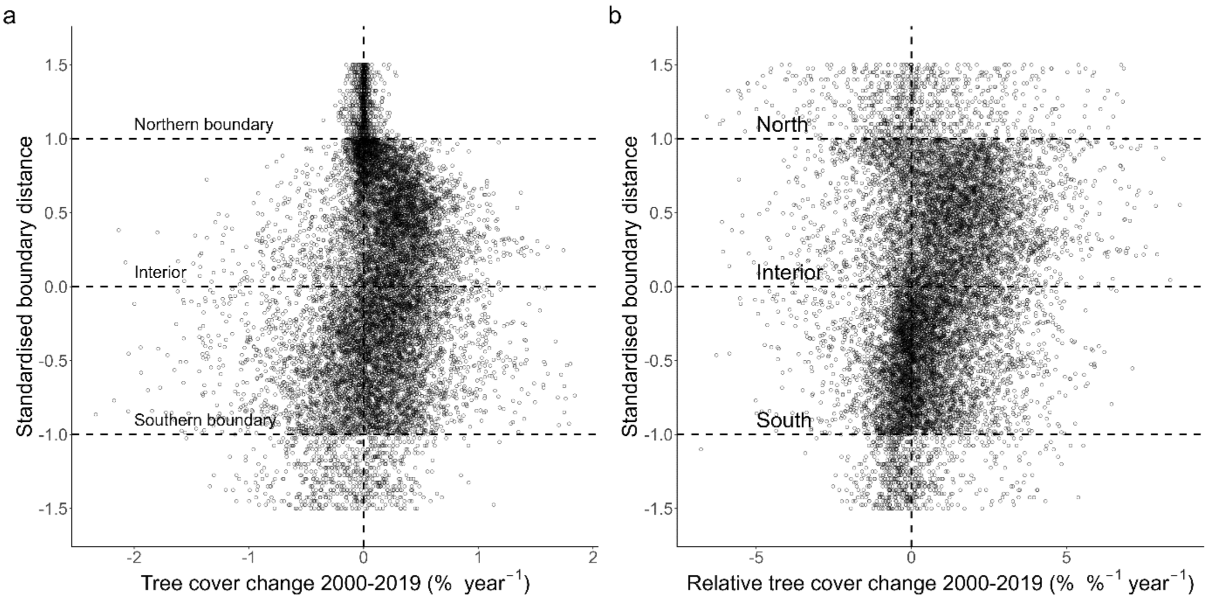

Figure 7 Tree cover changes of North American boreal forests from 2000-2019. Absolute (**a**) and relative (**b**) tree cover changes are shown for each of 12,954 sample plots along south-north transects.

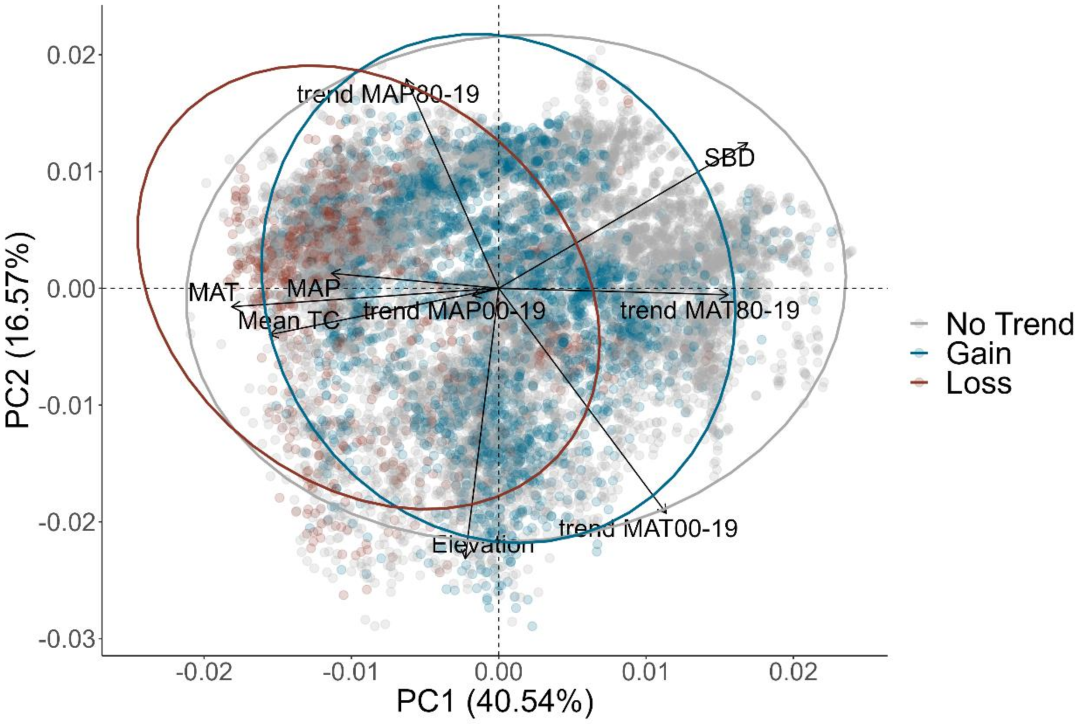

Figure 8 Principal component analysis of sample plots. The following variables are shown as vectors: mean tree cover (mean TC), boundary distance (SBD), elevation, mean annual temperatures (MAT), mean annual precipitation (MAP) and trends of temperature and precipitation over two periods (1980-2019 and 2000-2019). Point colours represent the direction of tree cover change: no significant trend, tree cover gains and tree cover losses. Significance terms were calculated from a Mann-Kendall-test using the 'zyp' package in R[2].

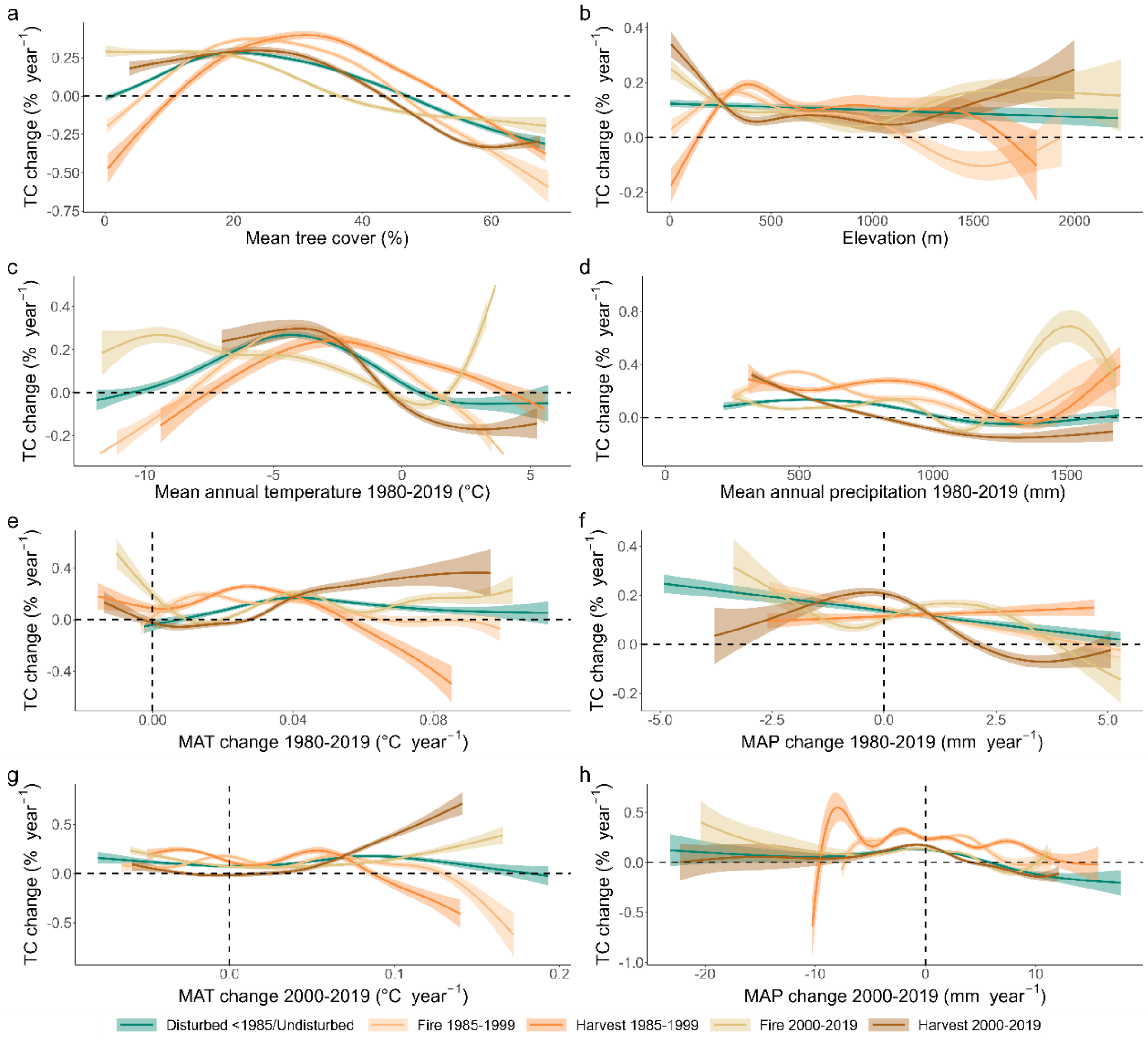

Figure 9 Relationships between absolute tree cover (TC) change 2000-2019 across North American boreal forests and environmental conditions: a Mean tree cover 2000-2019, **b** elevation, **c** mean annual temperatures (MAT), **d** mean annual precipitation (MAP), **e** change in MAT 1980-2019, **f** change in MAP1980-2019, **g** change in MAT 2000-2019 and **h** change in MAP 2000-2019. Relationships are shown for different disturbance categories (colours). Lines represent model fits ±standard errors around the fit from generalised additive mixed-effects models. Each predictor of tree cover change was included in a separate model due to correlations between predictors. Transects were used as random effects in the models. Additionally, tree cover change was fitted by disturbance type and assuming an exponential spatial correlation structure. Fitted model lines including raw data points are shown in Figure 18. Absolute tree cover change was calculated through a Theil-Sen's slope estimation. Standardised boundary distance represents the distance to the southern and northern boreal biome boundary, i.e. -1 = South (S), 0 = Interior (I), 1 = North (N). Boundaries were derived from Gauthier et al. 2015[1]. Climatic variables were taken from the ERA5 Monthly Averaged data set and cover the period 1980-2019 (2000-2019 for recent climate change trends).

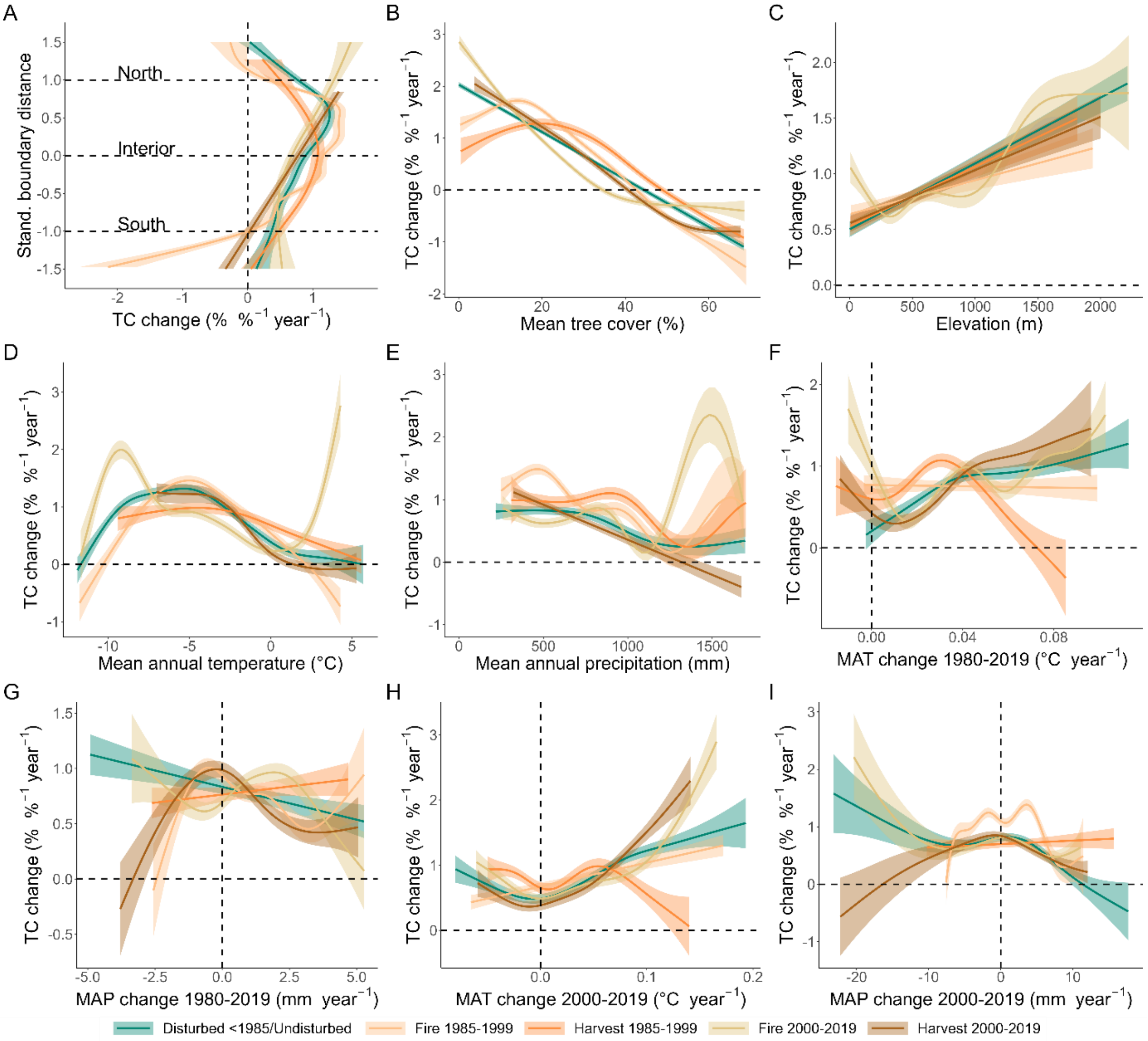

Figure 10 Relationships between relative tree cover (TC) change 2000-2019 across North American boreal forests and environmental conditions: (A) standardised boundary distance, (B) mean tree cover 2000-2019, (C) elevation, (D) mean annual temperatures (MAT), (E) mean annual precipitation (MAP), (F) change in MAT 1980-2019, (G) change in MAP1980-2019, (H) change in MAT 2000-2019 and (I) change in MAP 2000-2019. Relationships are shown for different disturbance categories (colours). Lines represent model fits ±standard errors around the fit from generalised additive mixed-effects models. Each predictor of tree cover change was included in a separate model due to correlations between predictors. Transects were used as random effects in the models. Additionally, tree cover change was fitted by disturbance type and assuming an exponential spatial correlation structure. Fitted model lines including raw data points are shown in Figure 19. Relative tree cover change was calculated through a Theil-Sen's slope estimation method and is in relation to mean tree cover across the study period. Standardised boundary distance represents the distance to the southern and northern boreal biome boundary, i.e. -1 = South (S), 0 = Interior (I), 1 = North (N). Boundaries were derived from Gauthier et al. 2015[1]. Climatic variables were taken from the ERA5 Monthly Averaged data set and cover the period 1980-2019 (2000-2019 for recent climate change trends).

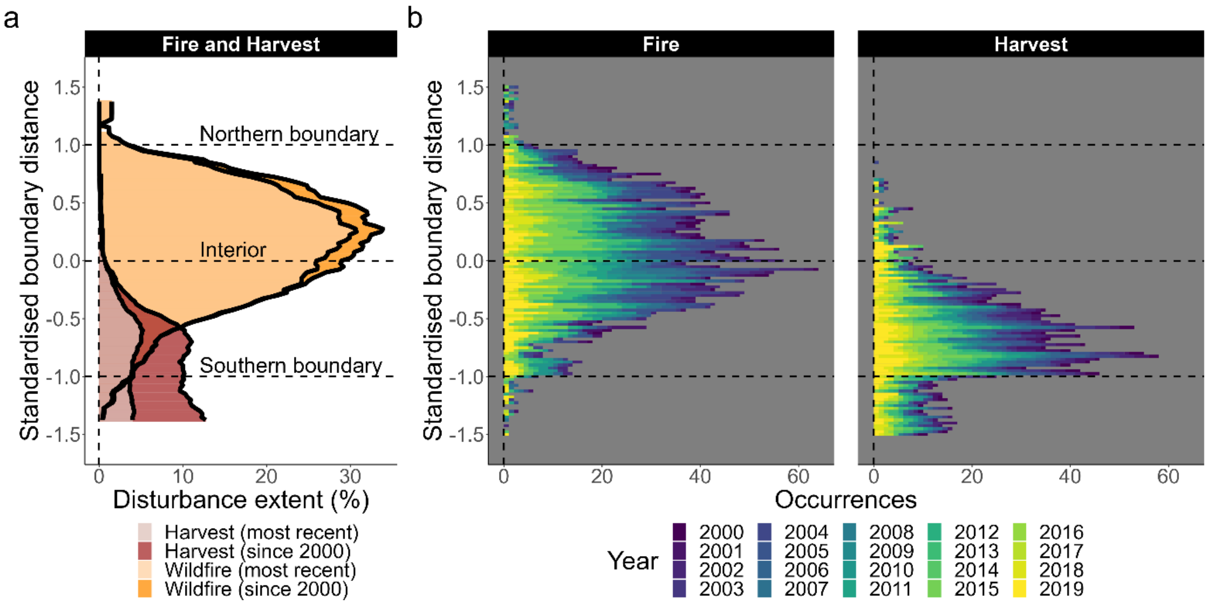

Figure 11 Spatial extent and occurrences of wildfire and timber harvest between 2000 and 2019 in relation to boreal boundaries. a Mean relative extent of sample plots which were disturbed. For each disturbance type, the extent is given for the most recent disturbance and accumulated for all disturbances since 2000. Extents are running means of binned boundary distances. **b** Accumulated number of sample plots disturbed by wildfire and timber harvest for each year between 2000-2019. Occurrences are summarised over binned boundary distances. Years are years of the largest and most recent disturbance recorded within sample plots.

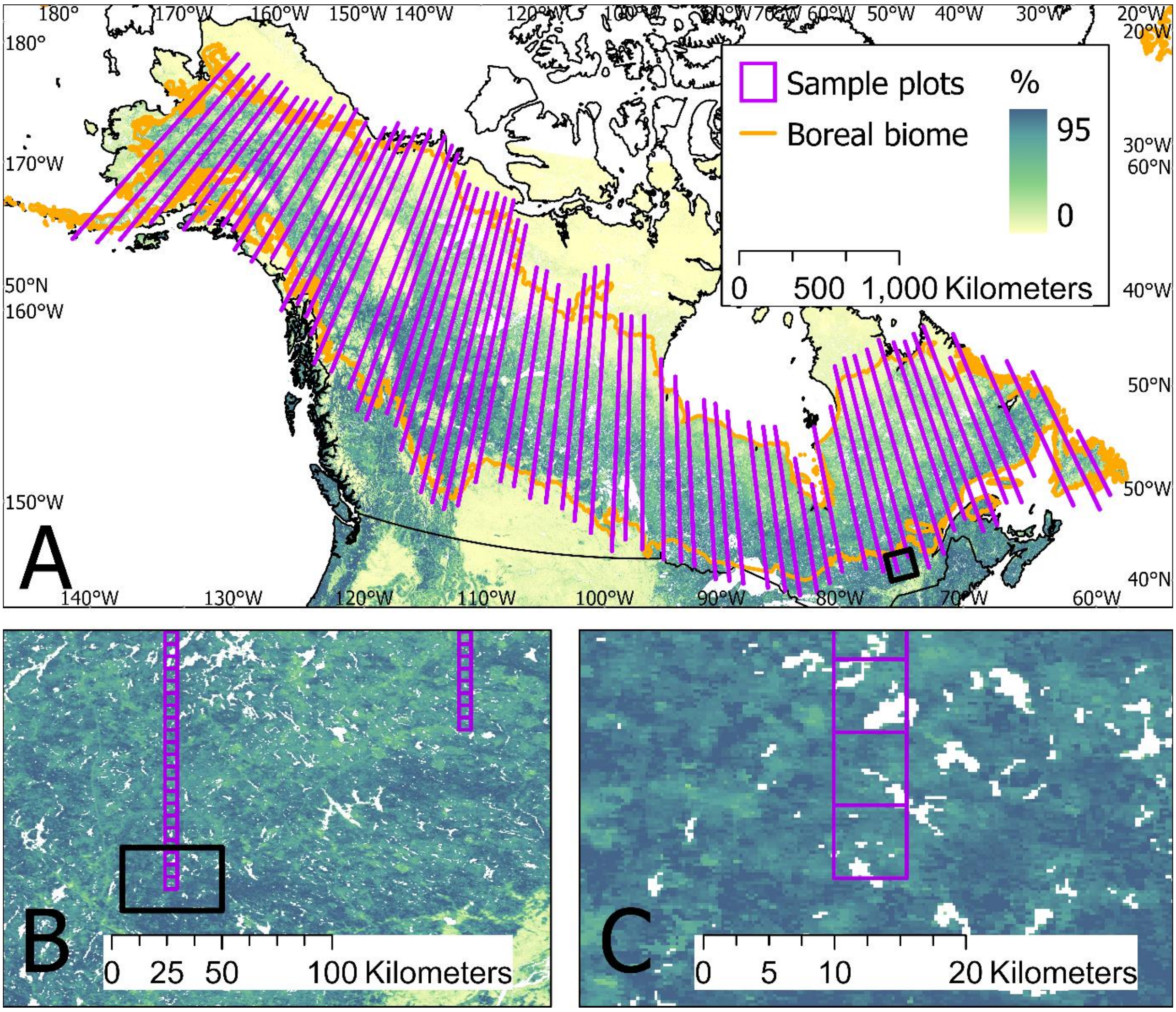

Figure 12 Sampling design with sample plots located along south-north transects across North America. Panels A-C show transects and sample plots with increasing spatial detail. Background colours represent MODIS tree cover estimates for the year 2019.

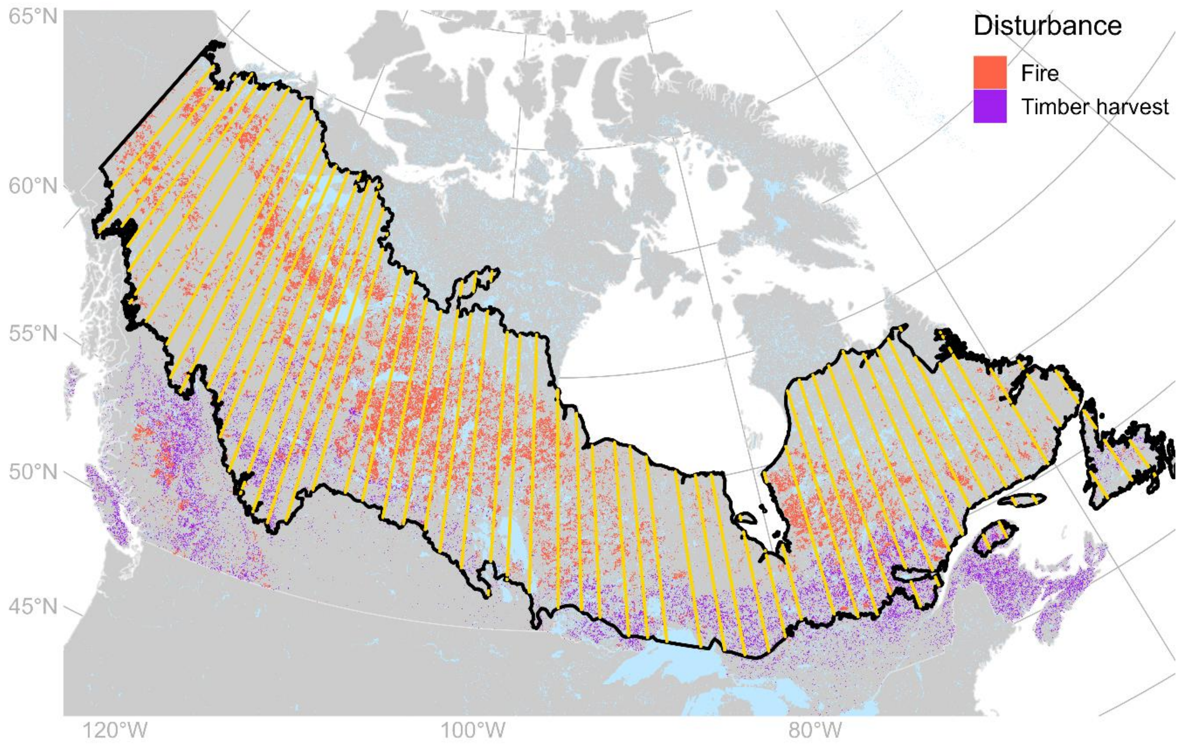

Figure 13 Occurrences of wildfire and timber harvest across the Canadian boreal biome based on the CanLaD disturbance dataset. The black line represents the biome extent. The yellow grids are sample plots within the biome we used in our study.

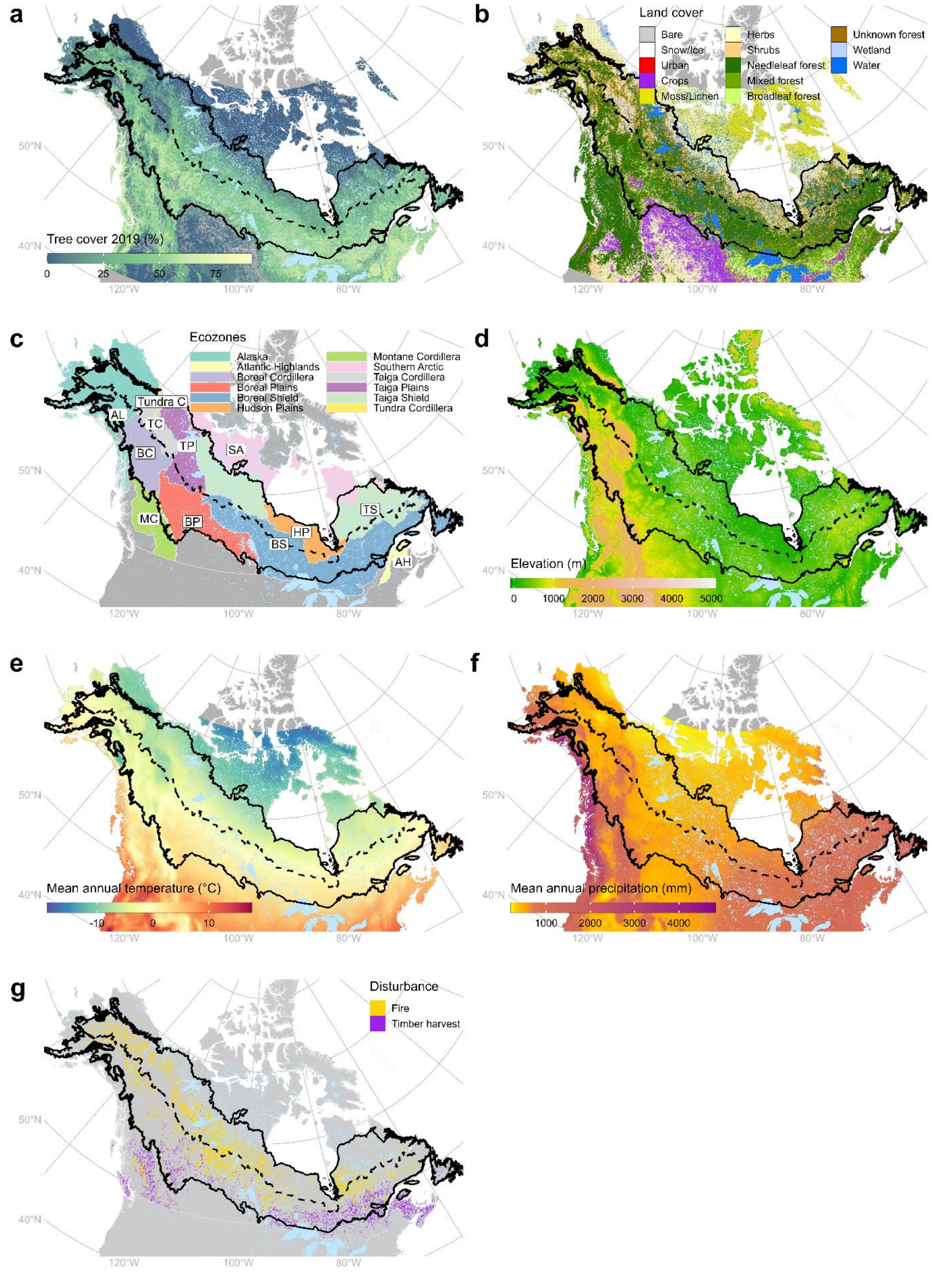

Figure 14 Overview of spatial data used in the analysis: **a** Tree cover, **b** land cover, **c** ecozones, **d** elevation, **e** mean annual temperature, **f** mean annual temperature and **g** Disturbances.

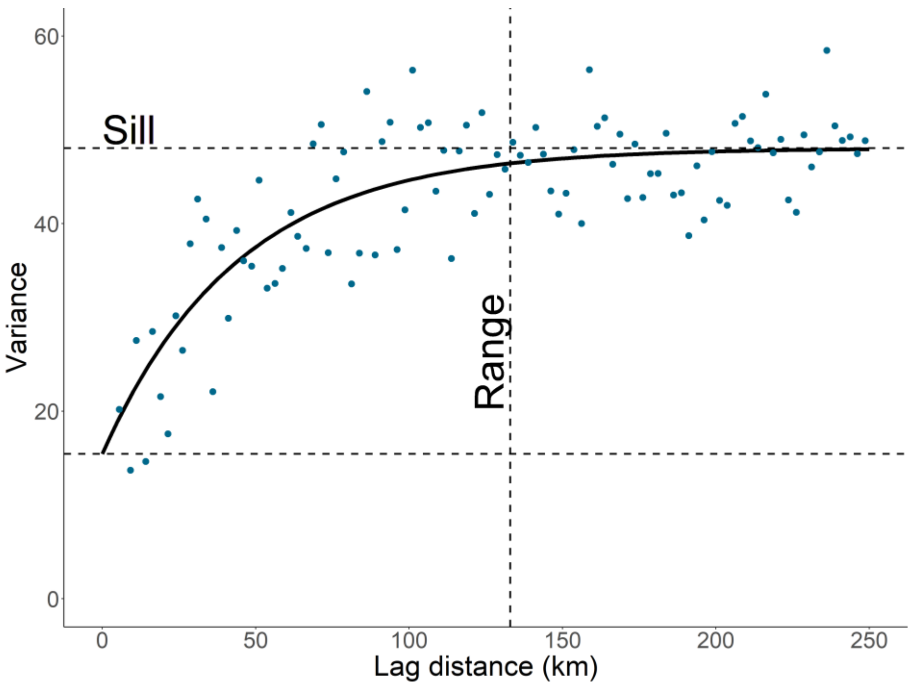

Figure 15 Variogram of tree cover trends. Variances were calculated for 10,000 random points across the North American boreal forests and binned into 100 distance groups (blue points). The bold line represents a fitted exponential variogram function. Dashed lines show the sill of maximum variance, the range at which variances and thus autocorrelated effects level off, and the nugget (the intrinsic variance for distances of 0, lower horizontal line).

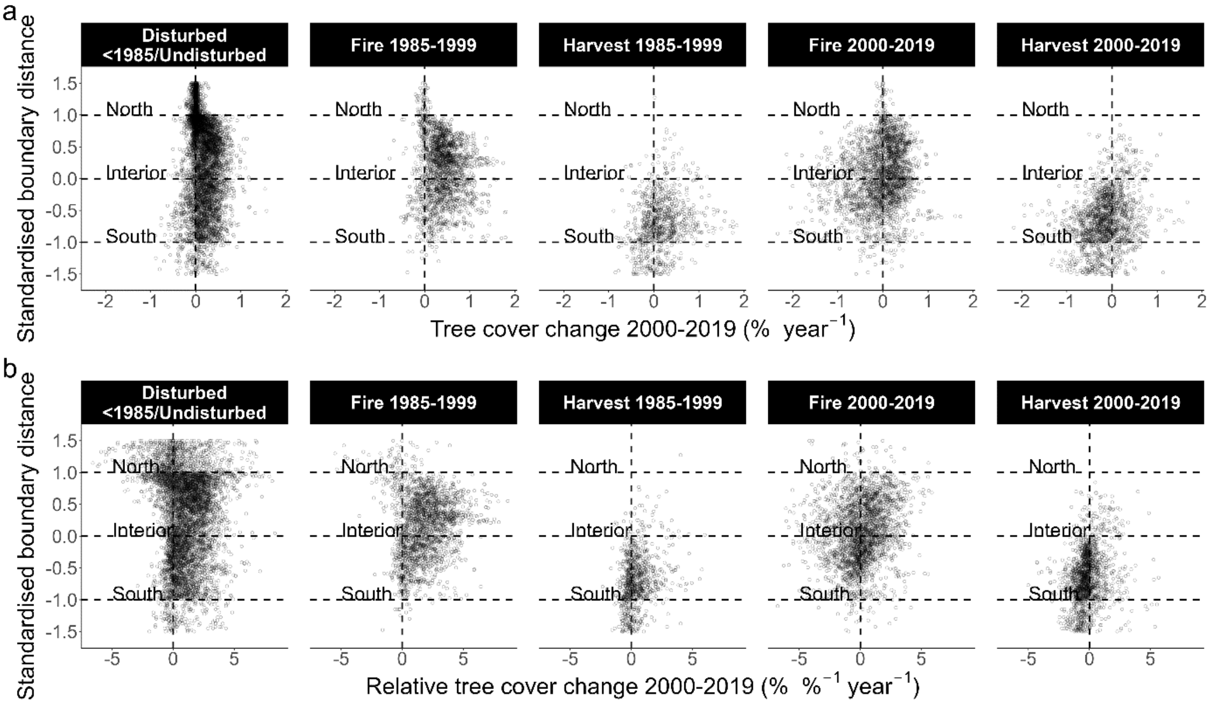

Figure 16 Tree cover changes between 2000-2019 separated by disturbance type. Absolute (**a**) and relative (**b**) tree cover changes are shown for each of 12,954 sample plots and for each disturbance type.

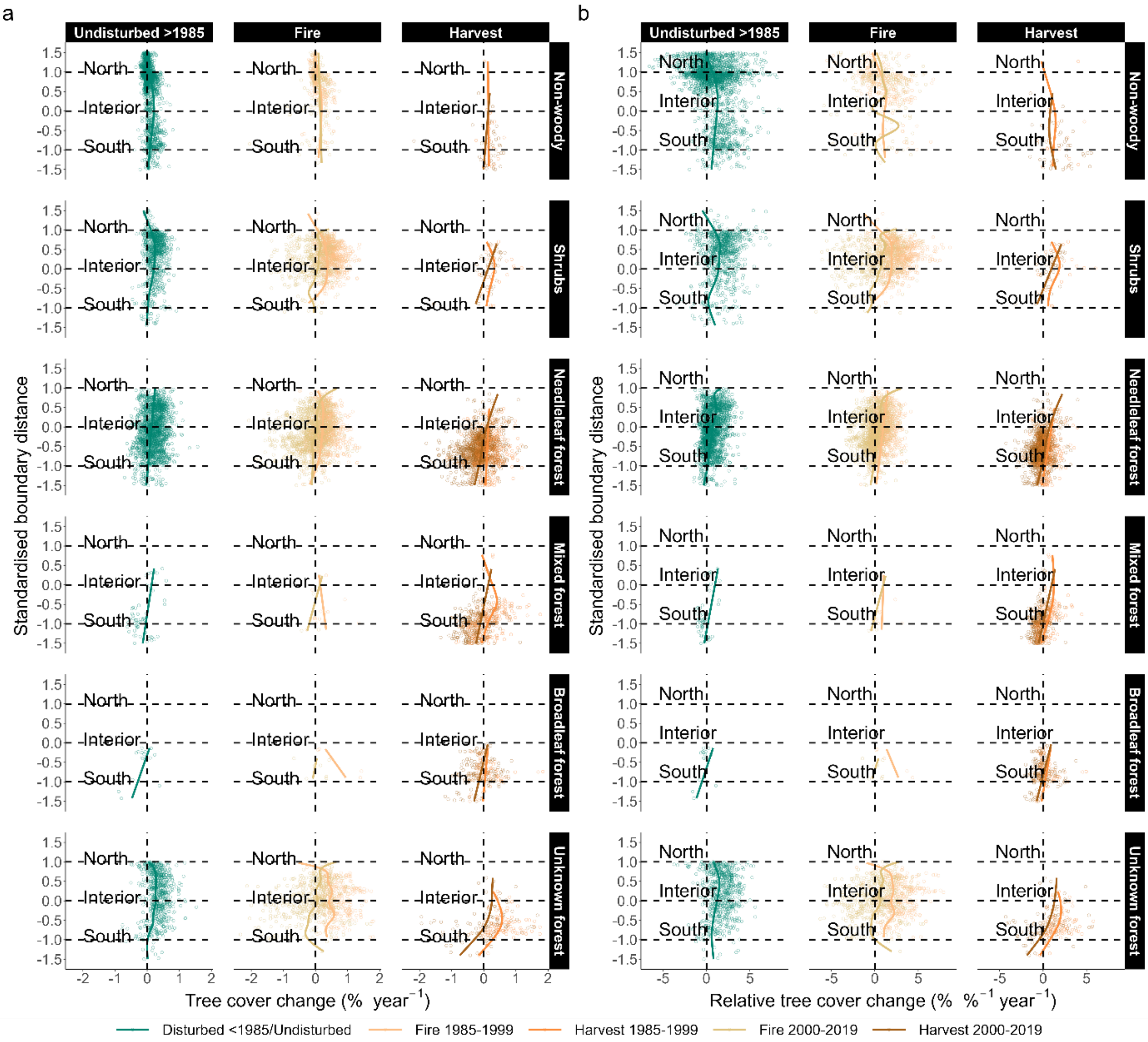

Figure 17 Relations between tree cover change and land cover type along the southern and northern boreal forest biome boundaries. Relationships are shown for absolute (**a**) and relative (**b**) tree cover changes. Plots are separated by six land cover types and include the values from sample plots and fitted lines. Colours represent different disturbance types.

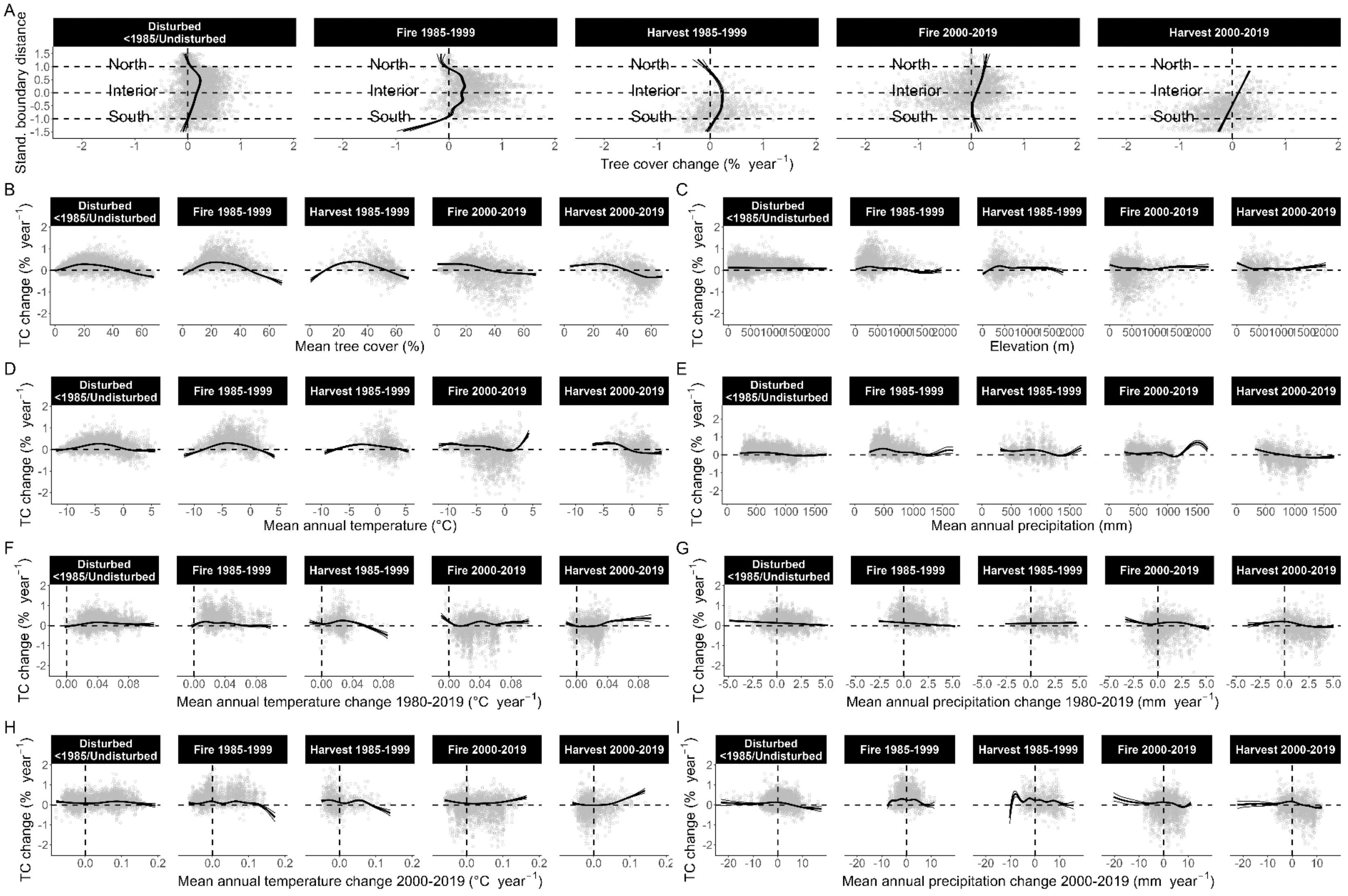

Figure 18 Relationships between absolute tree cover (TC) change 2000-2019 across North American boreal forests and environmental conditions. The model fit lines and environmental variables are the same as in Figure 10. Plots are separated by disturbance type and include the values from sample plots (grey points).

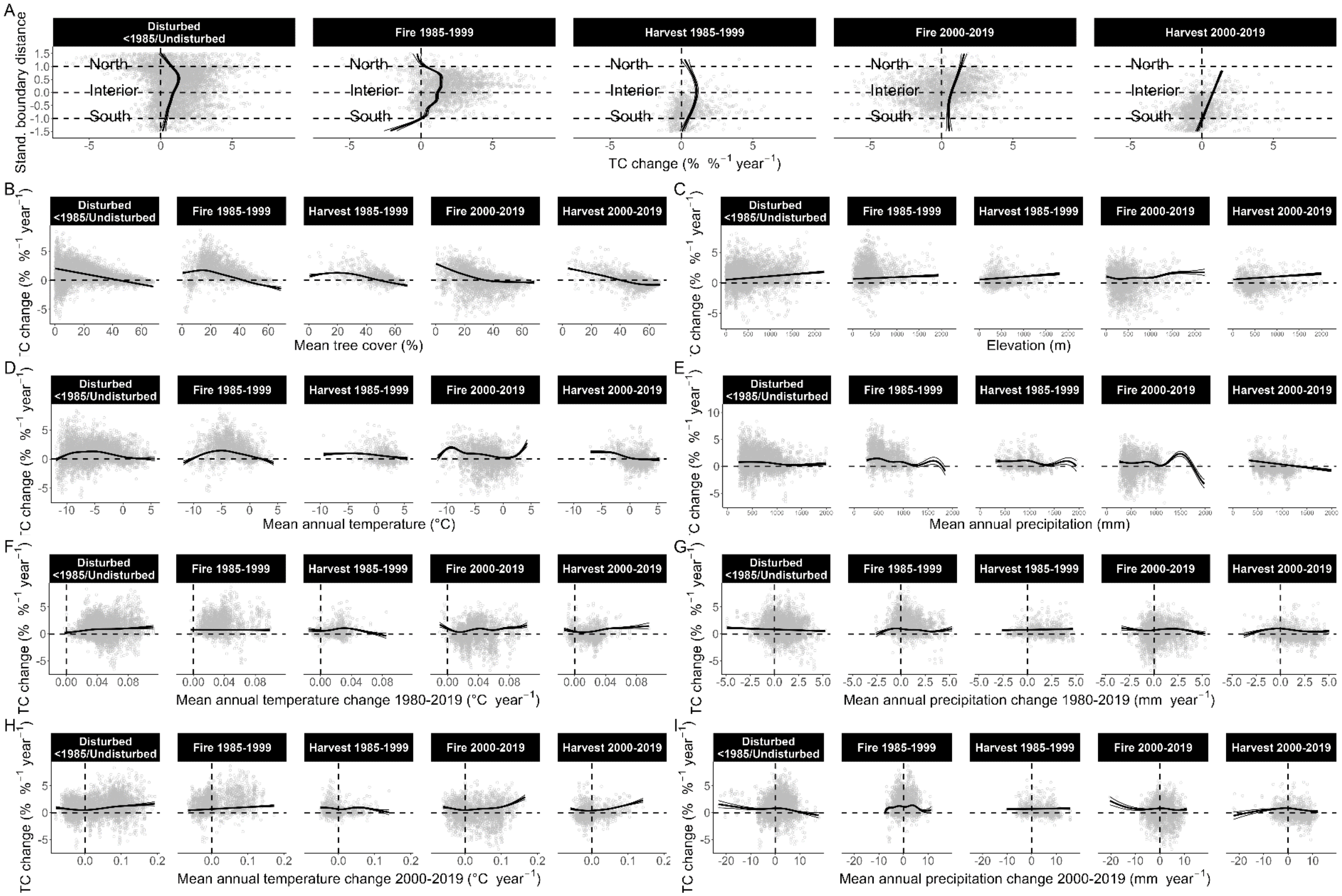

Figure 19 Relationships between relative tree cover (TC) change 2000-2019 across North American boreal forests and environmental conditions. The model fit lines and environmental variables are the same as in Figure 11. Plots are separated by disturbance type and include the values from sample plots (grey points).

Supplementary tables

Table 1 Area extent and proportion of fires and timber harvests within the Canadian boreal biome and the sample plots used in our study. The total area represents the area of the entire Canadian boreal biome and the sample plots. The proportion of disturbances within our sample plot is very similar to that of the entire Canadian biome.

	Canadian boreal	Sample plots
	Area (ha)	
Fire	58,633,211	1,917,950
Harvest	15,877,704	545,202
Total	529,482,711	17,251,036
	Proportion (%)	
Fire	11.1	11.1
Harvest	3.0	3.2

Table 2 Description and details of data sets on tree cover estimates and environmental variables.

Data set	Time period	Temporal resolution	Spatial resolution
MODIS VCF version 6 fractional tree cover	2000-2019	annual	250m
Copernicus global landcover map (GLC) version 2.0.2	2015	na	100m
ECMWF Re-analysis (ERA) version 5 monthly averaged climate data	1980-2019 and 2000-2019	monthly	0.25°
USGS Global Multi-resolution Terrain Elevation Data	2010	na	~250m
Canada Landsat Disturbance (CanLaD) including wildfire and timber harvest	1985-2019	annual	30m
Monitoring Trends in Burn Severity (MTBS)	1985-2019	annual	30m

Table 3 Reclassification of land cover classes from the Copernicus global land cover map[3] to 14 classes for this study.

Original class names	New class names
No input data available	No data
Shrubs	Shrubs
Herbaceous vegetation	Non-woody
Cultivated and managed vegetation/agriculture (cropland)	Crops
Urban / built up	Urban
Bare / sparse vegetation	Bare
Snow and Ice	Snow and Ice
Permanent water bodies	Water
Herbaceous wetland	Wetland
Moss and lichen	Non-woody
Closed forest, evergreen needle leaf	Needleleaf
Closed forest, evergreen, broad leaf	Broadleaf
Closed forest, deciduous needle leaf	Needleleaf
Closed forest, deciduous broad leaf	Broadleaf
Closed forest, mixed	Mixed forest
Closed forest, unknown	Unknown forest
Open forest, evergreen needle leaf	Needleleaf
Open forest, evergreen broad leaf	Broadleaf
Open forest, deciduous needle leaf	Needleleaf
Open forest, deciduous broad leaf	Broadleaf
Open forest, mixed	Mixed forest
Open forest, unknown	Unknown forest
Open sea	Ocean

Supplementary references

1. Gauthier, S., Bernier, P., Kuuluvainen, T., Shvidenko, A. Z. & Schepaschenko, D. G. Boreal forest health and global change. *Science* **349**, 819–822 (2015).

2. Bronaugh, D. & Werner, A. zyp: Zhang + Yue-Pilon Trends Package. Preprint at (2019).

3. Marcel Buchhorn *et al. Copernicus Global Land Service: Land Cover 100m: version 2 Globe 2015: Product User Manual (Version Dataset v2.0, doc issue 2.20)*. (2019).
