# Supplementary material for: Northern expansion is not compensating for southern declines in North American boreal forests
Source: Nat Commun. 2023 Jun 8;14:3373. doi: 10.1038/s41467-023-39092-2 (PMC10250320; doi:10.1038/s41467-023-39092-2)
Supplement: Supplementary file 3 — Description of additional supplementary files [file 41467_2023_39092_MOESM3_ESM.docx]

# Northern expansion is not compensating for southern declines in North American boreal forests

Description of additional supplementary files

**First and corresponding author:** Ronny Rotbarth

**Co-authors:** Egbert H van Nes, Marten Scheffer, Jane Uhd Jepsen, Ole Petter Laksforsmo Vindstad, Chi Xu, Milena Holmgren

File name: **Supplementary data 1**

Description: Raw data table including all variables used in data analyses. Included are variable values for each sample plot (in rows).

File name: **Supplementary data 2**

Description: Results from all generalised additive mixed-effects models establishing the relationships between tree cover change and environmental variables using the mgcv package in R. Each model represents the relationship between one predictor (independent) variable and tree cover change. Transects were used as random effects in the models. Additionally, tree cover change was fitted by disturbance type and assuming an exponential spatial correlation structure. Model analyses for all models were performed using the F-statistic as a one-sided test.
