# Supplementary material for: Northern expansion is not compensating for southern declines in North American boreal forests
Source: Nat Commun. 2023 Jun 8;14:3373. doi: 10.1038/s41467-023-39092-2 (PMC10250320; doi:10.1038/s41467-023-39092-2)
Supplement: Supplementary file 6 — Reporting Summary [file 41467_2023_39092_MOESM6_ESM.pdf]

nature portfolio

Corresponding author(s): Ronny Rotbarth
Last updated by author(s): May 1, 2023

Reporting Summary

Nature Portfolio wishes to improve the reproducibility of the work that we publish. This form provides structure for consistency and transparency in reporting. For further information on Nature Portfolio policies, see our Editorial Policies and the Editorial Policy Checklist.

Statistics

For all statistical analyses, confirm that the following items are present in the figure legend, table legend, main text, or Methods section.

n/a	Confirmed	
☐	☒	The exact sample size (n) for each experimental group/condition, given as a discrete number and unit of measurement
☐	☒	A statement on whether measurements were taken from distinct samples or whether the same sample was measured repeatedly
☐	☒	The statistical test(s) used AND whether they are one- or two-sided<br>*Only common tests should be described solely by name; describe more complex techniques in the Methods section.*
☐	☒	A description of all covariates tested
☐	☒	A description of any assumptions or corrections, such as tests of normality and adjustment for multiple comparisons
☐	☒	A full description of the statistical parameters including central tendency (e.g. means) or other basic estimates (e.g. regression coefficient) AND variation (e.g. standard deviation) or associated estimates of uncertainty (e.g. confidence intervals)
☐	☒	For null hypothesis testing, the test statistic (e.g. F, t, r) with confidence intervals, effect sizes, degrees of freedom and P value noted<br>*Give P values as exact values whenever suitable.*
☒	☐	For Bayesian analysis, information on the choice of priors and Markov chain Monte Carlo settings
☒	☐	For hierarchical and complex designs, identification of the appropriate level for tests and full reporting of outcomes
☐	☒	Estimates of effect sizes (e.g. Cohen's d, Pearson's r), indicating how they were calculated

Our web collection on statistics for biologists contains articles on many of the points above.

Software and code

Policy information about availability of computer code

Data collection	We used Esri's ArcPro, version 2.8.3 for extracting data from remote sensing products
Data analysis	We used R (version 4.2.0) in connection with R studio (version 1.1.463) for data analysis. We used the following R packages: (1) zyp, version 0.10-1.1, for quantifying temporal trends in tree cover, (2) mgcv, version 1.8-40, for building general additive mixed-effects models, (3) stats, version 4.2.0, for performing principal component analyses. The code used for data analyses is provided on the first author's github: https://github.com/RonRotbarth/BorealContraction

For manuscripts utilizing custom algorithms or software that are central to the research but not yet described in published literature, software must be made available to editors and reviewers. We strongly encourage code deposition in a community repository (e.g. GitHub). See the Nature Portfolio guidelines for submitting code & software for further information.

Data

Policy information about availability of data

All manuscripts must include a data availability statement. This statement should provide the following information, where applicable:

- Accession codes, unique identifiers, or web links for publicly available datasets
- A description of any restrictions on data availability
- For clinical datasets or third party data, please ensure that the statement adheres to our policy

Tree cover data extracted from MODIS VCF, Version 6 is available via the Application for Extracting and Exploring Analysis Ready Samples (AppEEARS, https://appeears.earthdatacloud.nasa.gov/). An updated CanLaD disturbance dataset on wildfire and timber harvest from 1985 to 2020 was kindly provided by Dr Luc

Guindon and will soon be available on the Government of Canada website (https://open.canada.ca/data/en/dataset/add1346b-f632-4eb9-a83d-a662b38655ad). ERA5 climatic data on surface temperatures and precipitation are available on the Copernicus website, DOI: 10.24381/cds.f17050d7. Elevation data is available on the USGS website (DOI: 10.5066/F7J38R2N) following the link to the GMTED2010 Viewer (https://topotools.cr.usgs.gov/gmted_viewer/viewer.htm). The global land cover map is available on Zenodo, DOI: 10.5281/zenodo.3243508. Boreal forest boundary data is available upon request to Sylvie Gauthier and Dominique Boucher. The map of tree cover trends produced by this study is available on an open repository, DOI: 10.5281/zenodo.7520322. All source data are provided with this paper in the supplementary materials.

Human research participants

Policy information about studies involving human research participants and Sex and Gender in Research.

Reporting on sex and gender	N/A
Population characteristics	N/A
Recruitment	N/A
Ethics oversight	N/A

Note that full information on the approval of the study protocol must also be provided in the manuscript.

Field-specific reporting

Please select the one below that is the best fit for your research. If you are not sure, read the appropriate sections before making your selection.

☐ Life sciences ☐ Behavioural & social sciences ☒ Ecological, evolutionary & environmental sciences

For a reference copy of the document with all sections, see nature.com/documents/nr-reporting-summary-flat.pdf

Ecological, evolutionary & environmental sciences study design

All studies must disclose on these points even when the disclosure is negative.

Study description	We studied the trend in tree cover over the past 20 years in North American boreal forests using remote sensing tree cover data. We analysed the relationship between tree cover trends and the following predictors: disturbances (wildfire 1985-1999, wildfire post 2000, timber harvest 1985-1999, timber harvest post 2000 and disturbed prior 1985), land cover types (Non-woody, shrubs, needleleaf forests, mixed forests, broadleaf forests and unknown forests), elevation, mean tree cover and climatic conditions (mean and trend in annual temperatures and precipitation). We used a set of 12,954 sample plots located along 69 randomly placed north-south transects.
Research sample	We sampled from a range of existing remote sensing datasets. The key dataset is a percentage proportion of tree cover within 250x250m pixels. This dataset covers two decades of tree cover (2000-2019) at moderate spatial resolution and is one of only a few datasets of tree cover estimates available on a large spatial scale (i.e. global coverage). Datasets of covariates are described in detail in the methods.
Sampling strategy	Within each sample plot, tree cover data (and covariates) were averaged for each study year (2000-2019) and used in further analyses. The total sum of sample plots was not pre-determined but depended on the number and location of randomly placed transects. Plots were aligned along these transects and the length of each transect thereby determined the number of sample plots per transect. The number of transects were chosen based on a spatial correlation analysis which revealed a minimum distance between transects at which spatial correlation would be expected to level off. That distance determined the number of transects randomly spread out across the North American boreal biome.
Data collection	We extracted and averaged data from each sample plot using standard geographical information software. The lead author performed data collection.
Timing and spatial scale	We took samples for all available years between 2000-2019 provided by the tree cover data set. For other data sets the individual time periods are stated in the methods. The spatial scale of 250m was accumulated
Data exclusions	Criteria for excluding some sample plots were made beforehand. We excluded sample plots that were located within specific land cover types, such as water features, urban or agricultural areas, as these did not represent the study system of boreal forests. We did not exclude any further data.
Reproducibility	Our study is not experimental. However, our study design can be replicated easily by following the instructions in our methods. Any other data we used were taken from existing and available datasets.
Randomization	We randomised the location of transects and thus sample plots. Consequently, sample plots were allocated randomly into groups based on the location-specific nature of disturbance regime or land cover type.
Blinding	Blinding does not apply to our observational study.

Did the study involve field work? ☐ Yes ☒ No

Reporting for specific materials, systems and methods

We require information from authors about some types of materials, experimental systems and methods used in many studies. Here, indicate whether each material, system or method listed is relevant to your study. If you are not sure if a list item applies to your research, read the appropriate section before selecting a response.

Materials & experimental systems

n/a	Involved in the study
☒	☐ Antibodies
☒	☐ Eukaryotic cell lines
☒	☐ Palaeontology and archaeology
☒	☐ Animals and other organisms
☒	☐ Clinical data
☒	☐ Dual use research of concern

Methods

n/a	Involved in the study
☒	☐ ChIP-seq
☒	☐ Flow cytometry
☒	☐ MRI-based neuroimaging
